# Employee Retention in the Service Industry in Malaysia

**DOI:** 10.3389/fsoc.2022.928951

**Published:** 2022-07-08

**Authors:** Nur Syafiqah Binti Zainal, Walton Wider, Surianti Lajuma, Mohd Wafiy Akmal B. Ahmad Khadri, Nasehah Mohd Taib, Asong Joseph

**Affiliations:** ^1^Faculty of Business and Communication, INTI International University, Nilai, Malaysia; ^2^School of Science & Psychology, International University of Malaya-Wales, Kuala Lumpur, Malaysia

**Keywords:** employee retention, work-life balance, work environment, reward and compensation, service industry, Malaysia

## Abstract

This study aims to investigate the effects of work-life balance, work environment, and reward and compensation on employee retention in Malaysia. A total of 400 questionnaires were collected online from employees within the service industry in Malaysia. Partial least square structure equation modeling was used to test the model and hypotheses. The results reveal that work-life balance and work environment had a strong positive effect on employee retention, but reward and compensation had a much stronger positive effect on employee retention. This research provides unique theoretical contributions by investigating these factors in the midst of the COVID-19 outbreak as components of the reciprocal process between employee and employer, and their effects on employee retention. This study also provides vital insights to business organizations to consider designing effective employee retention plans for a successful business.

## Introduction

A recurring difficulty faced by organizations today is the problem of increased employee turnover and the retention of employees (Al-Suraihi et al., 2021). In developing countries like Malaysia, employee turnover intention has become a serious problem (Munir and Tobi, 2020). Over the last decade, employee retention has become a key challenge for organizations as the demand for skilled employees has risen in businesses (Chakravarti and Chakraborty, 2020). Employers in Malaysia are dealing with high rates of voluntary turnover, for example, the voluntary turnover rate in 2017 was 12.8%, compared to a median of 10% in all major Asia-Pacific markets (Hewitt, 2017). According to Willis Towers Watson. (2017), within 2 years about 36% of employees in Malaysia were likely to leave their organization. In addition, based on Malaysia media reports, retention is indicated by a high turnover ratio, with up to 150,000 employees losing their jobs during the epidemic as a result of the financial crisis and lockdown (Ibrahim et al., 2021).

Research on employee retention highlights that the precise reasons why individuals quit organizations are complex and frequently linked to work-related stressors. Securing and keeping qualified employees play a crucial part in any organization because the knowledge and expertise of employees are essential for the economic competitiveness of the organization (Mathimaran and Kumar, 2017; Chakraborty and Biswas, 2020). Employees have become a priority since they positively contribute to the attainment of organizational goals and mission (Johennesse and Chou, 2017; Rattu and Tielung, 2018), long-term business health, and profitability (Aswale, 2017); therefore, companies must seek to understand why people stay or go (Bibi et al., 2018). Turnover intention is an employee's willingness to leave his current organization. The intention arises when there is a lack of motivation, promotion and performance in the workplace, which causes the employee to quit the job and leave the organization (Bhayo et al., 2017; Kaur and Randhawa, 2020). Finding the right person for the right position is difficult enough, but HR professionals face a considerably greater challenge in keeping them (Kamalaveni et al., 2019). Voluntary and involuntary turnover, on the other hand, are two different forms of turnover which are distinguished by whether the employee or the employer makes the decision to end the work relationship (Sun and Wang, 2017; Zheng et al., 2021). In contrast to voluntary turnover, a higher involuntary turnover may indicate issues with an organization's hiring strategy and processes. According to Birruntha (2019), 6.5% of the voluntary turnover attrition rate starts in the first half of the year across industries. In Malaysia, the consumer goods industry has the highest voluntary turnover rate at 8.4%. The Malaysian labor market is always growing, and high turnover rates show that retaining employees is a tough challenge (Kadiresan et al., 2019). Although the employee turnover issue is not new (Menon, 2020), considering the COVID-19 pandemic, corporations were facing a lot of problems in the retention of employees, hence, the current study is aimed at better understanding the potential factors influencing employee retention.

Several factors contribute to employee retention, namely, communication, reward programmes, career development, and performance-based bonuses, among other elements. This indicates that compensation, training, and performance appraisal all have a favorable effect on employee retention (Bibi et al., 2018). In order to retain employees, organizations must also provide a supportive work environment (Kundu and Lata, 2017; Naz et al., 2020). Employees today are different; they are not short of choices (Priya and Sudhamathi, 2019). If an employee is dissatisfied, they will leave and seek employment elsewhere; thus, the organization must work hard to retain its people; otherwise, it would lose its best employees (Aguenza and Som, 2018). The independent variables in this study are the reciprocal processes between employee and employer such as work-life balance, work environment, and reward and compensation. Therefore, this study aims to answer the research question, “What are the perceived influences of work-life balance, work environment, and reward and compensation on employee retention in the Malaysian service industry?” Answering this question is important because there are currently few studies on employee retention in the Malaysians service industry (Fauzi, 2019). It is hoped that the findings of this study will be useful to Malaysian companies in terms of designing a fully integrated retention plan, whilst taking into consideration COVID-19.

## Theoretical Underpinnings

The theoretical foundation of the study is social exchange theory (SET) (Blau, 1964). SET is an approved process of exchange that leads to relationship satisfaction linked to human connections (Shahsavarani et al., 2016). Blau's idea of social exchange is employed in a variety of scenarios to form people's attitudes and behaviors, as well as to guide the context of relationships (Diah et al., 2020). The reciprocal process between employees and their existing employers is the rationale for SET and the decision on turnover intentions (Osman et al., 2016). Furthermore, SET is the most commonly used theory for a deeper understanding of employee retention (Aman-Ullah et al., 2020). It is used to clarify the direct relationship between work-life balance and involvement in employee development activities (Blau, 1964). It suggests that employees who are contented with their work environment are more likely to stay with an employer for a longer period of time (Baharin and Hanafi, 2018), When workers perceive that their organizations are meeting their needs in the workplace, they show extensive commitment by responding with increased enthusiasm in performing tasks and exhibiting preferred service ethics, mannerisms, behaviors, and attitudes (Kumar et al., 2018). To put it another way, when employees feel driven and favored, they are more likely to have a greater effect in the workplace (Baharin and Hanafi, 2018). Moreover, when employees perceive that organizations value their wellbeing, they engage in organizational citizenship behavior. Such perceived organizational support increases the expectation that a greater commitment to organizational goals will be rewarded, thus increasing employee commitment to the organization, and enhances employee retention (Shah and Asad, 2018). SET assumes that all components of the environment are a portion of a characterized social structure (Diah et al., 2020). One of the edifying ways to illustrate an employee's dependability on the company is extending commitment in the right way through positive behavior, mental behavior, and extraordinary effectiveness (Pisano et al., 2017).

Furthermore, SET gives a perfect framework for understanding the relationship between organizations and their employees (Diah et al., 2020). This exchange controls the orientation and interface of the employees and communicates the sort of results they will get (Jung and Takeuchi, 2019). Regardless of whether the employee focus is economic or social, it can be used to leverage this aspect of employee culture and act as inspiration for increased efficiency and long-term results (Newman et al., 2019). Hence, it is envisaged that employee advancement initiatives will boost employee commitment to the organization as well as their motivation to work (Diah et al., 2020). SET may help to explain why employees participate in committed behavior without expecting personal reward or legal compliance, as well as why organizations value commitment and loyalty (Cropanzano et al., 2017). Positive activities that assist the organization's personnel produce a favorable working environment (Diah et al., 2020). People are an organization's most precious asset, and a sufficient support structure is required to allow them to grow and develop their abilities to their full potential (Tiwari, 2017). Employees require proper growth opportunities, such as assessment, professional advancement, and positive interpersonal interactions (Diah et al., 2020). Employees who feel irrelevant as a result of poor development efforts are more likely to abandon and change behavior, which harms a company's performance goals and its growth cycle (Childs et al., 2017).

Investing in employee development is a prerequisite for organizational effectiveness and efficiency, according to SET, because it positively impacts employees' recognition of new skills and professional requirements, as well as their level of commitment and motivation to achieve organizational goals (Diah et al., 2020). Modern organizations are more flexible in their recruitment and compensation strategies and they now welcome friend referrals, which makes working relationships more personal and intimate (Chernyak-Hai and Rabenu, 2018). Furthermore, trust in the organization's ability to reward employees for achieving their exchange commitments is connected to organizational support (Zhao et al., 2021). An employee satisfied with the compensation provided by the company will be willing to keep working for a long time (Ali and Anwar, 2021). However, the self-centeredness of individuals is emphasized in SET, and exchange actions can appear to be self-centered and selfish (Blau, 1964). To put it another way, an individual will perform an evaluation on the potential rewards gained by interacting with others first, and social exchange will not take place if neither of the two sides receives adequate compensation (Yin, 2018). As a consequence, employees will strive to strike a mutually beneficial balance in exchange relationships and sustain a long-term positive social exchange connection at work; it holds that the purpose of human action is to maximize benefits while minimizing costs.

## Literature Review

### Employee Retention

Retention is defined as the ability to maintain an employee's relationship with a company (Kadiresan et al., 2019) and is essential for success in today's business world (Baharin and Hanafi, 2018). Employee retention is crucial because it allows companies to gain a competitive advantage and serves as a visible representation of goals met (Bakar et al., 2018). Retention is also considered significant because it has an impact on the company's efficiency in terms of monetary and non-monetary values (Kadiresan et al., 2019). The process in employee retention refers to the policies and practices that businesses use to keep important employees from leaving, including taking steps to encourage employees to stay with the company for as long as feasible (Baharin and Hanafi, 2018). Many companies nowadays are looking at employee retention measures by reaching out to their employees to ensure their job satisfaction and keep them at the company for as long as possible (Mahadi et al., 2020). This is linked to the company's attempts to help its employees, evidenced by the number of people who leave or join the organization (Tian et al., 2020). Retaining helps human resource planning by anticipating the gap between future workforce demand and supply based on the organization's goals (Kamalaveni et al., 2019). When employees have some voice and authority over their job decisions, they are more likely to stay (Khalid and Nawab, 2018). The key factors that contribute to turnover intention and employees' main considerations are compensation, supervisor support, and work-life policies (Kamalaveni et al., 2019). Additionally, human behavior is reinforced by contextual elements such as the environment and nature of the job (Subramaniam et al., 2019).

Broadening and adopting retention strategies are encouraged to be used to build up organizations and keep them well-equipped to deal with issues of employees who want to leave or quit (Kadiresan et al., 2019). Successful retention includes more than what a company does once an employee has been employed and established within the company (Kumar and Kavitha, 2018). Employees who earn a pay rise as a result of their outstanding performance on the job are more likely to feel satisfied and accomplished in the long run (Subramaniam et al., 2019). Those who are satisfied and comfortable with their jobs, are more engaged in their work and continually strive to increase their organization's customer satisfaction objective (Menon, 2020). When the younger generation enters the workforce amid constrained economic and budgetary circumstances, organizations require strategies and procedures such as employee participation to retain competent people (Khalid and Nawab, 2018). Organizations must ensure that adequate measures are being taken to prevent employees from leaving their jobs (Yao et al., 2019). The cost of replacing a worker is often 2.5 times an individual's pay, thus, majority of corporations are willing to put in effort to preserve working conditions, allowing the organization to keep current employees (Kaur, 2017; Kaur and Randhawa, 2020). A study therefore is needed to determine the effect of work-life balance, work environment, and reward and compensation on employee retention in the Malaysian context, with a focus on the service industry.

## Hypothesis Development

### The Influence of Work-Life Balance on Employee Retention

Work-life balance is often used by human resources as a management tool to increase employee work satisfaction and engagement (Welmilla, 2020). A well-planned work-life balance will help with some issues, including worker health, contentment, and motivation, while also lowering employee turnover. Seeking work-life balance does not mean employees do not want to work as much, but that they want the flexibility to complete their tasks on time while still having time to rest; nevertheless, this flexibility for the worker is still missing in every industry (Adnan Bataineh, 2019). Work-life balance is becoming increasingly important in the business world, as work interruptions into employees' time have been shown to negatively affect employees' attitudes toward their jobs. Further evidence has shown that work interference in family life damages employees' work fulfillment and reduces their job commitment (Tan, 2020). When an organization encourages a work-life imbalance culture, such as work overload, employees' time with their families becomes limited, for example, they are not able to attend important events like family members' birthdays (Wong et al., 2017). A lack of work-life balance will have severe consequences for both employees and the organization (Marques and Berry, 2021). On the other hand, employees too need to understand how to divide their time between job and personal life. Work-life balance refers to a person's daily equitable distribution of work and leisure activities (Hee and Ann, 2019), and it is essential to focus on these five aspects of a working person's life: work, family, friends, health, and self (Agha, 2017; Sagayadoro et al., 2021). Therefore, a family-friendly policy is beneficial as it improves employees' work-life balance by providing flexible work hours or reducing working hours so that more time can be spent with their families.

Neglecting the work-life balance of employees will result in poor outcomes in employee performance, as well as bring repercussions to the organization. Many believe that work-life balance entails more than just having time to spend with their families; they want a company to also value them as individuals and treat them with respect. According to Choi (2020), flexible working schedules may have an impact on workers' desire to stay in their jobs, satisfaction, and commitment, because employees who are burdened with long work hours often struggle to fulfill the needs of their families. Work-Life policies assist employees to better manage their work and family lives, as well as improve attitudes and behaviors such as organizational attachment, job satisfaction, and intention to stay. Hashim et al. (2016) reported a high quality and direct relationship between work-life balance and retention among middle management employees in Malaysia. Additionally, research by Adriano and Callaghan (2020) on professionals in part-time study in South Africa showed that a favorable work-life balance could help to improve job satisfaction and employee overall turnover intention. Therefore, we hypothesized that:

*H1: Work-life balance positively affects employee retention*.

### The Influence of Work Environment on Employee Retention

The physical geographical location of the workplace, as well as its immediate surroundings, such as construction sites or office buildings, are all part of the work environment (Rattu and Tielung, 2018). Environmental factors are a collection of all aspects that affect the environment where an employee is located and might have an effect on the job satisfaction of the employee (Abun et al., 2021). According to Hee and Rhung (2019), the work environment is another motivator for employees while at the workplace. Esthi (2020) pointed out that the working environment can play a significant role in influencing whether or not an employee stays with the organization. According to Haldorai et al. (2019), employee turnover is substantially higher when they work in a poor environment, because employees feel that their efforts are not recognized and appreciated by others. A positive work environment will lead to positive and higher employee performance and thus, high employee retention (Naz et al., 2020). An employee performs more easily and faces fewer challenges in a pleasant working environment (Nugroho and Suryani, 2021). Employees prefer to work in a friendly, clean, and convenient environment, and they can achieve significantly higher productivity in such a setting. A positive, compassionate and enjoyable work environment is one in which employees have a say in decisions that affect their work-life balance (Subramaniam et al., 2019). Rattu and Tielung (2018) stated that employees are said to feel better about coming to work in a positive work environment, which provides the incentive they need to get through the day.

Additionally, a healthy work environment will develop with a continuous learning culture where skills increase at a consistent rate (Hee and Rhung, 2019). But, when the work environment is constrained and inadequate, employees will feel uneasy and perform poorly. They will experience negative consequences such as low motivation and engagement, as well as stress and burnout, if their work environment and job design are not rewarding and meaningful (Subramaniam et al., 2019). Suifan et al. (2018) found that supervisory support is significant in physically expressing and signaling a supportive work environment in a company. Employees are also likely to be more engaged in their work when an organization provides new technology and tools to improve the working environment (Feige, 2019; Chan et al., 2021). Therefore, the current study hypothesized that:

*H2. Work environment positively affects employee retention*.

### The Influence of Reward and Compensation on Employee Retention

Many businesses and organizations use a variety of incentives or rewards to motivate staff to improve their performance (Sittenthaler and Mohnen, 2020). Reward can also be defined as a variety of one-of-a-kind advantages delivered to employees as a substitute for effort or non-trivial monetary value (Choi and Presslee, 2020). Primary rewards include financial remuneration, benefits, and other non-cash compensation, as well as job experience (Kulikowski and Sedlak, 2020). There are many definitions of rewards, but basically they are benefits received in exchange for effort and worth. According to Kryscynski et al. (2021), incentive consists of different forms, such as monetary rewards, recognition and awards, physical services, and benefits that organizations provide for their employees. Monetary rewards can also be in the form of tangible rewards, which include both direct rewards such as basic salary, incentives, and stocks, as well as indirect rewards such as extra benefits like vacation, insurance and pension (Kassahun, 2021). Even more, a high-potential employee should be paid more than the industry average in order to encourage them to stay with the company (Singh, 2019). The different kinds of rewards are to incentivize and retain employees, and ensure higher productivity (Feige, 2019). Employees who have been identified and rewarded by the organization are more connected and eager to stay and do their utmost for the company's success (Tan, 2020). Rewards can also be a direct way to motivate staff to work toward the company's ultimate objective (Kršlak and Ljevo, 2021). Meanwhile, employees with low pay are less likely to engage in practices such as delegation, consultation, and suggestion, resulting in low employee retention among first-line workers (Khalid and Nawab, 2018).

Research conducted in Malaysia found that the highest types of rewards for employees were pay and fringe benefits (Seman and Suhaimi, 2017). Employee benefits and benefits in kind (fringe benefits, perquisites, and perks) are non-wage compensations given to employees in addition to their regular pay (Boella and Goss-Turner, 2019). The importance of pay in attracting and retaining employees has been known for decades, and it is becoming increasingly important in today's competitive economic environment, where strategic compensation planning is required (Arokiasamy, 2019). Such an environment requires companies and corporations to strategise their compensation structures, tailoring to the individual and catering to intrinsic and extrinsic rewards (Vizano et al., 2021). Moreover, retention approaches like communication, reward programmes, career development, performance-based bonuses and compensation are elements that have shown a positive impact on employee retention (Bibi et al., 2018). For instance, in order to retain employees, companies must understand their needs so that an effective compensation and reward system can be established to fulfill them (Oduntan, 2020). The most essential components of compensation are salary, merit pay and bonuses, all of which are designed to attract the best employees. Compensation and rewards practices in the organization have made employee retention successful (Ardiansyah et al., 2020). Such incentives should be designed to meet the requirement for employees to be appropriately compensated for their contributions, efforts, and abilities (Rai et al., 2019). It seems apparent that encouraging a healthy work-life balance is not the only method to keep employees; good reward management in a competitive range, career and development possibilities, and a variety of other elements all play an important role (Kamalaveni et al., 2019) in attracting, retaining, and motivating people to do their jobs effectively for the benefit of the company (Jam and Jamal, 2020). Therefore, we hypothesized that:

*H3. Rewards and compensation positively affects employee retention*.

## Methods

### Population and Sample

In order to measure and study the targeted sample of this research, the first step is to determine the estimated number of the target population who are employees that are working in the service industry in Malaysia. According to Hirschmann (2021), the number of employees in Malaysia is ~9.2 million in 2020 overall. The sample frame selected to conduct this study included service industry employees in the categories of senior management, middle management, entry level, internship, and others who, during the data collection, were aged between 20 and 50. G^*^Power was used to determine the minimum required sample size in terms of statistical power (Faul et al., 2009). The model of this study had three predictors. By using G^*^Power with an effect size of 0.15, alpha of 0.05, and a power of 0.95, the minimum sample size needed was only 119. Thus, we can safely say that our study with a sample size of 400 has a power of more than 0.95 and is large enough, and the findings can be used with confidence. The data collection was conducted using online survey *via* social networking sites to recruit participants using a non-probability snowball sampling method. This procedure takes samples that are either related to or referred to by earlier samples (Taherdoost, 2016). In this research, snowball sampling was chosen because this technique helped gain representation from various levels, backgrounds, genders and age groups, and covered a wide geographical area (Asiamah et al., 2017).

### Data Collection Procedure

Data collection was held over a 2-week period in November 2021. Unfortunately, due to the COVID-19 outbreak, people were afraid to make close contact everywhere they go, especially in the workplace and public areas. Research activities were affected, requiring a slew of procedural changes. Thus, transitioning to online data collecting was deemed necessary to ensure compliance with safety requirements. Using a snowball method, the first participant who met the inclusion criteria was chosen to complete the self-report survey, which was distributed *via* the internet (link created from google form) through social networking sites such as WhatsApp, Facebook, and LinkedIn. The respondent was then asked to recommend other people in the sample frame who would be good candidates to take the survey. A total of 400 questionnaires were received in which all were valid to be used for further analysis.

Table 1 illustrates the summary of the demographic profile of the 400 respondents. In the age categories, the majority of respondents were aged between 30 and 39 (37.2%, *n* = 149), followed by 40–49 years of age (34.5%, *n* = 138), and 20 to 29 (16.8%, *n* = 67), while in the minority were the 50 years and above age group (5.2%, *n* = 21) and the below 20 years age group (6.3%, *n* = 25). In terms of education level, the majority of respondents held a Bachelor's degree (54 %, *n* = 216), followed by respondents who had completed pre-university education (22.75 %, *n* = 91), and respondents who had completed secondary school education (11 %, *n* = 44). Only 8% of the respondents (*n* = 32) had a postgraduate degree, while 4.25 % (*n* = 17) had no formal education. In the length of service categories, 129 (32.25%) worked for 11–15 years, while 125 (31.25%) worked for 6–10 years, followed by 81 (20.25%) who worked for 3 to 5 years. A minority group of 27 (6.75%) respondents had worked in the organization for 16–20 years and another 38 (9.5%) worked for 2 years and below in their current organization. With regard to current job position, majority of the respondents were in middle management (55.25%, *n* = 221), followed by entry level (18.75%, *n* = 75), others (13.5%, *n* = 54), and senior management (11%, *n* = 44), while a minority was in the internship (1.5%, *n* = 6) position.

**Table 1:** Demographic profile of respondents.

		**Frequency**	**%**
Age	Below 20 years	25	6.3
	20–29	67	16.8
	30–39	149	37.3
	40–49	138	34.5
Education level	50 years and above	21	5.3
	No formal education	17	4.3
	Secondary school	44	11.0
	Pre-University	91	22.8
	Bachelor's degree	216	54.0
	Postgraduate degree	32	8.0
Length of service	2 years and below	38	9.5
	3–5 years	81	20.3
	6–10 years	125	31.3
	11–15 years	129	32.3
	16–20 years	27	6.8
Position	Senior management	44	11.0
	Middle management	221	55.3
	Entry level	75	18.8
	Internship	6	1.5
	Others	54	13.5

### Measures

Employee retention: This construct comprises 5 items which were adapted from Jun et al. (2006) using the 5-Point Likert Scale, ranging from 1 (*strongly disagree*) to 5 (*strongly agree*). The following items were used: “I am prepared to put in a great deal of effort beyond what is normally expected in order to help my company to be successful,” “I am loyal to my company,” “This is the best company to work for,” and “I would recommend my company to a friend if he/she is looking for a job” and “I will choose this company if I was given a chance to choose again.” The Cronbach alpha coefficient was 0.86.

Work-life balance: This construct comprises 4 items adapted from Wong and Ko (2009) using the 5-Point Likert Scale, ranging from 1 (*strongly disagree*) to 5 (*strongly agree*). The following items were used: “I am satisfied with the time I spend at work and in my personal life, and privileges offered,” “I have good time management between work and personal life,” “I'm able to manage my work, family and life as the organization supports work-life balance,” and “I achieve balance and have enough time to spend on family duties and societal roles”. The Cronbach alpha coefficient was 0.92.

Work environment: This construct comprises 5 items adapted from Siddiqi (2011) and Njuguna and Owuor (2016) using the 5-Point Likert Scale, ranging from 1 (*strongly disagree*) to 5 (*strongly agree*). The following items were used: “I am satisfied with the physical working conditions in my company,” “I am satisfied with my work hours,” “My company utilizes new technologies at work,” “The working environment in my company is comfortable,” and “The duration of time to complete a work task assigned is reasonable in my company.” The Cronbach alpha coefficient was 0.81.

Reward and compensation: This construct comprises 4 items adapted from Hoole and Hotz (2016) using the 5-Point Likert Scale, ranging from 1 (*strongly disagree*) to 5 (*strongly agree*). The following items were used: “My company respects every employee and make each of us feel important,” “I am satisfied with the rewards given for achievements attained,” “Rewards and compensations given by the organization motivate me to perform better,” and “The organization's overall compensation and reward system is fair and equitable and thus, motivates me to work harder.” The Cronbach alpha coefficient was 0.86.

### Data Analysis

A partial least squares—structural equation modeling (PLS-SEM)—was applied to assess the measurement model and structural model, and test developed hypotheses. The PLS-SEM approach was adopted because the study was prediction-oriented research which aimed to predict factors that could influence the retention of employees in the service industry. The SmartPLS 3.0 software package was used to perform PLS-SEM.

## Results

### Assessment of Measurement Model

This section indicates the criteria necessary to confirm the reliability and validity of the measurement model. A total of 400 samples were used to assess both the measurement and structural models. The measurement model used in this study comprises four reflective constructs, namely, employee retention (ER), work-life balance (WLB), work environment (WE), and reward and compensation (RC). The assessment of reliability involves determining the value of outer loadings, composite reliability (CR) and Cronbach's alpha (CA), which should be >0.70. The assessment of convergent validity is by confirming the average variance extracted (AVE) to be >0.5 (Hair et al., 2019). Two items (ER1 and WLB1) were removed due to factor loadings below 0.70. The results of the assessment of measurement model in Table 2 shows acceptable values of CR and CA of all constructs in this study. Furthermore, all item loadings were greater than the value of 0.7, which, assuming that the CR and AVE met the required thresholds, is acceptable (Hair et al., 2019). All constructs had an AVE above 0.5, which illustrates an acceptable degree of convergent validity.

**Table 2:** Results of measurement model assessment.

**Latent variable**	**Items**	**Loading**	**AVE**	**CR**	**CA**	**Mean**	**SD**
Employee retention	ER2	0.86	0.828	0.951	0.930	3.14	0.82
	ER3	0.926					
	ER4	0.932					
	ER5	0.921					
Work-life balance	WLB2	0.936	0.880	0.956	0.932	3.13	0.84
	WLB3	0.940					
	WLB4	0.938					
Work environment	WE1	0.908	0.766	0.942	0.923	3.08	0.79
	WE2	0.864					
	WE3	0.843					
	WE4	0.876					
	WE5	0.884					
Reward and compensation	RC1	0.898	0.854	0.959	0.909	2.99	0.89
	RC2	0.932					
	RC3	0.932					
	RC4	0.934					

*AVE, Average Variance Extracted; CR, Composite Reliability; CA, Cronbach Alpha; SD, Standard Deviation*.

Hair et al. (2019) also recommended the establishment of discriminant validity during the assessment of reflective measurement models. There are two common ways to ascertain discriminant validity, namely, the Fornell-Larcker criterion and the heterotrait-monotrait (HTMT) ratio (Rasoolimanesh and Ali, 2018). However, the HTMT criterion has been established as the more conservative approach compared to more traditional assessment methods such as the Fornell-Larcker criterion (Henseler et al., 2015). Discriminant validity is established when the value of HTMT is <0.90 (Ali et al., 2018). Table 3 shows the value of HTMT for all constructs is lower than 0.90, therefore establishing discriminant validity.

**Table 3:** Discriminant validity using HTMT ratio.

**Constructs**	**ER**	**RC**	**WE**	**WLB**
ER				
RC	0.856			
WE	0.833	0.827		
WLB	0.804	0.734	0.897	

*ER, Employee Retention; WLB, Work-life Balance; WE, Work Environment; RC, Reward and Compensation*.

### Assessment of Structural Model

According to Table 2, the mean scores and standard deviations (SD) for our study variables were 3.14 for employee retention (SD = 0.82); 3.13 for work-life balance (SD = 0.84); 3.08 for work environment (SD = 0.79); and 2.98 for reward and compensation (SD = 0.89). To ensure no lateral collinearity issue in the structural model, the collinearity between research variables was examined (Hair et al., 2017). Table 3 shows that all inner VIF values were below 5 (Hair et al., 2017), indicating that there is no collinearity issue among the predictor constructs in the structural model.

**Figure 1:**
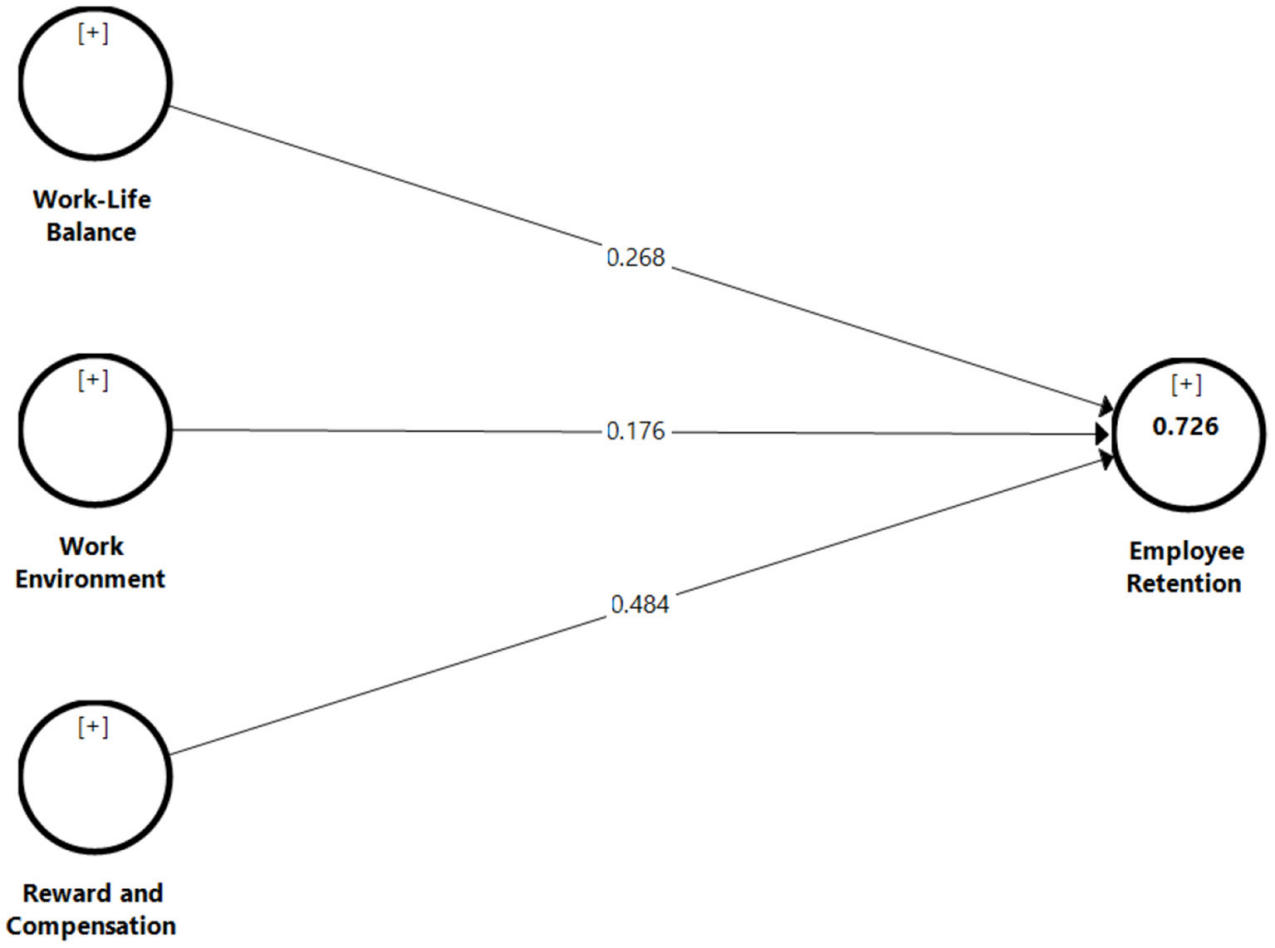
Results of assessment of structural model.

In order to assess the structural model, the *R*-squared (*R*^2^) value for the endogenous latent variable, the significance of path coefficients using the *p*-value and a confidence interval of 95% (CI 0.95), and the effect size (f ^2^) have to be assessed and reported (Hair et al., 2017). The values of 0.726 for the *R*^2^ of employee retention is considered high in behavioral studies (Hair et al., 2017), suggesting that 72.6% of the variance for employee retention can be described by work-life balance, work environment, and reward and compensation. The next step in the assessment of the structural model involves hypothesis testing by reporting the direct effect using the product coefficients approach (Nitzl, 2016). Table 4 and Figure 1 indicate the results of the hypotheses testing, including the path coefficients and the effect size for each path. The values of the effect size should be >0.02 (Cohen, 1988). To determine the path coefficient's significance, resampling techniques (e.g., bootstrapping) can be used. The path coefficients of the structural model were assessed using the bootstrap method with 5,000 resamples as suggested by Hair et al. (2017). The results show positive and direct effect of work-life balance (β = 0.268, *t* = 5.024, *p* < 0.01), work environment (β = 0.176, *t* = 2.864, *p* < 0.05), and reward and compensation (β = 0.484, *t* = 9.146, *p* < 0.01) on employee retention, with the highest effect being on reward and compensation and lowest effect on work environment. Therefore, H1, H2, and H3 were verified. Another important criterion to assess the predictive capability of the model is Stone-Geisser's *Q*^2^ (Hair et al., 2017). A *Q*^2^ value greater than zero indicates predictive relevance. The results of cross validated redundancy indicate that the value of *Q*^2^ for employee retention is 0.594, which is considered very high to assess predictive reliance of the research model (Hair et al., 2017).

**Table 4:** Results of hypothesis testing.

**Hypothesis**	**Relationship**	**Coefficient**	***t*-value**	**95% CI**	***f* ^2^**	**Supported**	**VIF**
H1	WLB → ER	0.268	5.024	[0.162, 0.370]	0.079	Yes	3.296
H2	WE → ER	0.176	2.864	[0.057, 0.297]	0.026	Yes	4.311
H3	RC → ER	0.484	9.146	[0.383, 0.591]	0.337	Yes	2.538

*ER, Employee Retention; WLB, Work-life Balance; WE, Work Environment; RC, Reward and Compensation*.

## Discussion

The main goal of this study is to determine whether work-life balance, work environment, and reward and compensation affect employee retention in the service industry in Malaysia. All the hypotheses are accepted. Reward and compensation is the strongest indicator of employee retention, followed by work-life balance and work environment.

The findings of this study reveal that reward and compensation is the strongest predictor of employee retention in service industry in Malaysia, thus, supporting H3. Our findings corroborate those of previous studies by Arokiasamy (2019), Ardiansyah et al. (2020) and Oduntan (2020). Compensation and reward has always been identified as a key factor in recruiting and maintaining employees, and it is becoming much more important in today's competitive economic environment, where strategic compensation planning is required. Moreover, compensating employees fairly demonstrates that they are respected as workers and as human beings. People are more motivated to come to work when they feel valued. They are more motivated to do a good job as a result of improved company morale. Likewise, when employees are aware that incentives or commissions are offered, they are driven to greater achievements. Hence, compensation programmes such as bonuses and commissions become a focal point for achievement. To develop a retention strategy that addresses employee compensation, management should be able to create a total reward structure that includes more than just compensation. Compensation and benefits packages for employees should be lucrative enough to entice valued employees to remain in the organization. It is recommended that compensation management be implemented in every organization in order to improve performance and retain employees. Moreover, total rewards strategies such as providing a total rewards package to employees, recognizing and rewarding individuals and teams who achieve specific objectives, goals or other milestones must be applied not just to retain competent employees, but also to stay competitive. As a reward system to motivate employees to stay engaged and productive at work, a good incentives plan will combine monetary pay and other advantages with opportunities for professional growth and development. Additionally, create and customize compensation policies to suit the organization's needs, goals and availability of resources. Providing workers with financial and tangible benefits helps to improve productivity and employee retention.

The H1 of this study was that work-life balance positively affects employee retention. The finding was supported, and therefore aligned to that of Kamalaveni et al. (2019), Adriano and Callaghan (2020) and Choi (2020). Work- life balance appears to be an important component of corporate social responsibility, and results show that organizational leaders should be aware of their responsibilities to ensure that their staff are not working so hard that it interferes with their personal lives, as this may create medical concerns, and in turn affect morale, productivity, and job satisfaction. It has to be acknowledged that employees will have fewer health problems and absences if they are encouraged to look after themselves and find work-life balance. This will make organizations more effective during business hours, as employees want to be a part of their growth. In addition, work-life balance also helps to attract a valuable talent pool of new hires whilst increasing retention rates. Time and money will be saved in maintaining a high level of talented employees. Some retention strategies that organizations can adopt to balance personal and professional lives include flexible working hours, flexible working arrangements, enough resources, opportunities for training, accurate workload, providing enough breaks during the day, authorization to take vacations, career and sabbatical leave, giving bonuses, inviting employees' families to staff gatherings, incentives for wellness and good management practices. Companies should set fundamental operation hours to guide employees and enable them to organize their work accordingly. Human resources must also play a key part in determining the most productive working hours for employees and make themselves more accessible, even introducing the flexible hours that are becoming more common among businesses today. Employees, especially the younger generation, may find this appealing because they appreciate the flexibility, and will strive to be more effective during working hours. It is a good idea to have feedback or work-life balance surveys from time to time. This is a crucial component of developing a healthy workplace. Situations change over time, and keeping track of these changes is critical if effective solutions are to be implemented. Employers can use these solutions to address issues that may be hindering the achievement of a good work-life balance. Hence, getting employee feedback and conducting surveys allows organizations to learn more about them and address the issues they are facing.

The last finding of this study which is related to H2 also reveals the significant effect of work environment on employee retention in the Malaysian service industry, thus confirming those of previous studies by Hee and Rhung (2019); Ni et al. (2020); Chan et al. (2021). A positive work environment is one of the most important factors influencing employee retention. Working in a friendly, clean and convenient environment is desirable, and allows employees to significantly increase productivity. The physical environment of the office or workplace, as well as the occupational health and safety of the employees, all contribute to a healthy work environment. It is important to always examine workplace safety to alleviate employees' concerns. For instance, one issue that employees should not have to be concerned about is whether all electric cables are covered or taped down with a cable tray to prevent people from tripping over them. Also, nobody likes to sit in a room with a sagging ceiling. Organizations are thus encouraged to provide a safe working environment for their employees. They should also promote a positive work environment by narrowing the concept of “occupational health and safety” and providing employees with training in workplace hazards and risks. Furthermore, employee training and family-friendly policies that offer flexibility in the workplace, such as when, where, and how an employee works, help to build a healthy work environment which favorably impacts employees' decisions to stay. New technologies should also be embraced in the workplace as the younger generation (Millennials and Gen Z) are global citizens, who not only accept new technologies but also welcome them in the workplace. Furthermore, as a result of the immediacy of digital media, corporate culture and businesses are becoming global entities. They must be technology savvy in order to have a global identity. When people from various regions, cultures, customs, and social structures come together, they bring with them new and distinctive ideas. They provide new perspectives on issues as well as more problem-solving options. And the end result is a much-needed global perspective on issues at hand. As new technologies promote the development of a strong work culture which, in the long term, benefits the company, it is a pathway that should be followed by all modern businesses.

### Limitations and Future Research

With the help of social exchange theory, the present study lends support to previous studies as well as clarifies the roles played by work-life balance, work environment, and reward and compensation in employee retention in Malaysia. Research Topics that are available in the theoretical field regarding the reciprocal process between employee and organization have become warranted in these recent years, though research that focuses on the Malaysia service industry is relatively insufficient. Therefore, from a theoretical point of view, this study has bridged the knowledge gap on building an ideal framework on employee retention. The findings will benefit future researchers who could take it further.

Several practical implications have also emerged. In today's fast-paced business world, organizations must continually adapt to change in order to remain competitive, and one way to do so is to promote a long-term retention plan for their employees. It is more challenging than ever, as employers are often forced to give more benefits or incentive packages as people often seek positions that provide the financial security they require in difficult economic times, especially during this pandemic period. The younger generation, on the other hand, is continuously on the hunt for professions that allow them to manage work and family life. They desire autonomy as well as the opportunity to make a big difference and contribution in the workplace. This study intends to help the service industry's management level take the necessary steps to retain employees based on the findings. The results can help organizations design an effective retention plan by giving employees more opportunities to be promoted, providing more incentive plans and compensation packages, more flexible time, and supportive and positive work environments to make employees felt more appreciated. These benefits and opportunities will ensure employees remain in the organization, thus helping it save on the cost of hiring new employees.

There are several limitations that must be considered in this research. First, data collection could only be done *via* online as Malaysia was under the Movement Control Order at that time. The time horizon for a similar research can be performed based on longitudinal studies instead of cross-sectional. Additionally, the current data was collected during the COVID-19 pandemic where unemployment rate and inflation are increasing, hence, replicative studies are warranted. Second, although this is a diverse sample, the participants are only from the service industry and this has limited the generalisability of the findings and researchers from outside Malaysia need to interpret the findings cautiously.

Future research could focus on diverse industries, such as manufacturing, technology or banking, to add more value to the research. If time had permitted, a larger sample could have been collected to increase the accuracy and validity of the research results. Several other influencing factors could be investigated in future studies to provide a more comprehensive understanding of culturally embedded viewpoints on employee retention.

## Conclusion

This study is greatly significant in that it offers insights into the reciprocal process of employees and organizations on employee retention in the context of the Malaysian service industry. In light of this, our study has revealed an ideal framework for understanding the relationship between organizations and their employees, focusing on crucial factors like work-life balance, work environment. Reward and compensation in particular is found to have the greatest impact. Nevertheless, as most companies in Malaysia are still pressured by the impact of COVID-19, it is important for these business organizations to take appropriate measures and make effective decisions to overcome these challenges in order to successfully implement an integrated retention plan. Our results also resonate with the rationale of SET which focuses on the reciprocal process between employees and their existing employers on employee retention. Our study, notably, has enriched this model by examining economic and social factors and identifying them as the forces that drive employee retention.

## Data Availability Statement

The raw data supporting the conclusions of this article will be made available by the authors, without undue reservation.

## Ethics Statement

Ethical approval was not provided for this study on human participants because the study was conducted according to the guidelines of the Declaration of Helsinki and following academic ethics. The patients/participants provided their written informed consent to participate in this study.

## Author Contributions

All authors listed have made a substantial, direct, and intellectual contribution to the work and approved it for publication.

## Conflict of Interest

The authors declare that the research was conducted in the absence of any commercial or financial relationships that could be construed as a potential conflict of interest.

## Publisher's Note

All claims expressed in this article are solely those of the authors and do not necessarily represent those of their affiliated organizations, or those of the publisher, the editors and the reviewers. Any product that may be evaluated in this article, or claim that may be made by its manufacturer, is not guaranteed or endorsed by the publisher.
